# Unprecedented Cell-Selection Using Ultra-Quick Freezing Combined with Aquaporin Expression

**DOI:** 10.1371/journal.pone.0087644

**Published:** 2014-02-18

**Authors:** Yasuhiro Kato, Takayuki Miyauchi, Youichiro Abe, Dušan Kojić, Manami Tanaka, Nana Chikazawa, Yuhki Nakatake, Shigeru B. H. Ko, Daisuke Kobayashi, Akihiro Hazama, Shoko Fujiwara, Tatsuya Uchida, Masato Yasui

**Affiliations:** 1 Department of Pharmacology, School of Medicine, Keio University, Tokyo, Japan; 2 Department of Systems Medicine, Sakaguchi Laboratory, School of Medicine, Keio University, Tokyo, Japan; 3 Department of Physiology, Fukushima Medical University School of Medicine, Fukushima, Japan; 4 Department of Life Science, Tokyo University of Pharmacy and Life Science, Tokyo, Japan; The Ohio State University, United States of America

## Abstract

Freezing is usually used for preservation and storage of biological samples; however, this process may have some adverse effects such as cell membrane damage. Aquaporin (AQP), a water channel protein, has been suggested to play some roles for cryopreservation although its molecular mechanism remains unclear. Here we show that membrane damage caused by ultra-quick freezing is rescued by the expression of AQP4. We next examine if the expression of AQP combined with ultra-quick freezing can be used to select cells efficiently under freezing conditions where most cells are died. CHO cells stably expressing AQP4 were exclusively selected from mixed cell cultures. Having identified the increased expression of AQP4 during ES cell differentiation into neuro-ectoderm using bioinformatics, we confirmed the improved survival of differentiated ES cells with AQP4 expression. Finally we show that CHO cells transiently transfected with *Endothelin receptor* A and *Aqp4* were also selected and concentrated by multiple cycles of freezing/thawing, which was confirmed with calcium imaging in response to endothelin. Furthermore, we found that the expression of AQP enables a reduction in the amount of cryoprotectants for freezing, thereby decreasing osmotic stress and cellular toxicity. Taken together, we propose that this simple but efficient and safe method may be applicable to the selection of mammalian cells for applications in regenerative medicine as well as cell-based functional assays or drug screening protocols.

## Introduction

Cryopreservation, a critical step in regenerative as well as reproductive medicine, has been only empirically related to cell type and freezing conditions [1]–[5]. Dumont *et al.* reported that cell viability is related to cooling rates [3].

Under low cooling rates (slow freezing), solutes migrate towards regions containing: unfrozen extracellular water, causing dehydration as intracellular water slowly migrates to balance a more concentrated external solution [1]–[5].

Most mammalian cells are frozen using DMSO as a conventional cryoprotectant under the low cooling rate of −1°C/min. However mouse ES (mES) cells and undifferentiated human ES (hES) have poor survival rate after slow freezing, because of apoptosis [6]. The molecular mechanisms of apoptosis are related to Rho-associated kinase (ROCK) and reactive oxygen species (ROS). Treatment with Y-27632, which is a specific inhibitor of ROCK, improved the survival rate of ES cells and induced pluripotent stem (iPS) cells in case of conventional slow freezing [7], [8].

On the other hand, at high cooling rates (quick freezing), extensive intracellular super-cooling and the formation of intracellular ice crystals usually occur, which causes an injury to the plasma membranes [1]–[5]. An alternative way to cryopreserve a variety of cell types, vitrification, has been previously attempted using human ES, however, potential contamination risks combined with its limited utility [9], [10]. Vitrification as well as ultra-quick freezing also requires the extremely high concentrations of cryoprotectants for Ice-free condition, which may cause cell membrane damage, probably due to toxicity of cryoprotectants as well as high osmotic shock. These problems prompted for development of simpler, more efficient, and reliable vitrification methods.

Recent studies demonstrated the roles of aquaporins (AQPs), a family of water channel proteins selectively permeated by water [11], in cryopreservation of mouse oocytes [12], microorganisms [5], [13], and on other sections. The expression of AQP3 improved the survival rate of mouse oocytes after cryopreservation. Furthermore, it has been demonstrated that the inhibition of AQP3 increases the sensitivity of prostate cancer cells to cryotherapy [14]. The overexpression of AQY1 and AQY2 in Saccharomyces cerevisiae obtained freeze-tolerance [15]–[17]. These observations coherently suggest that AQPs may play some roles in freeze-tolerance.

Here, we attempted to engage the cryoprotective effect of AQPs in the selection of specific mammalian cells, since only cells expressing AQPs have been shown as resistant to damage caused by freezing at high cooling rate [5]. Indeed, we successfully identified a freezing tolerance of mammalian cell lines with either exogenous or endogenous AQP expression. Furthermore, combined with bioinformatics, we demonstrated the possibility of selecting specific types of cells differentiated from embryonic stem (ES) cells when the cells express AQPs in the process of each differentiation stage [18]–[20], which can be applied to regenerative medicine. We also showed that co-transfection of a gene of interest with AQP results in efficient accumulation of cells expressing the gene product, upon multiple cycles of freezing/thawing, suggesting that this protocol would be a potential alternative for establishment of stable cell lines to perform functional assays or drug screening protocols.

## Materials and Methods

### Cell culture and transfection

Chinese hamster ovary (CHO) cells stably expressing human aquaporin-1 (AQP1) or mouse aquaporin-4 (AQP4) and Madin-Darby canine kidney cells (MDCK) cells stably expressing AQP4 were maintained at 37°C in a humidified atmosphere with 5% CO_2_-95% air in a growth medium consisting of Ham's F-12 (Wako, Japan) for CHO and D-MEM (Wako) for MDCK fortified with 10% FBS (fetal bovine serum) (Wako), 1% penicillin/streptomycin (Life Technologies) and 0.5 mg/mL G418 antibiotics (Nacalai tesque) in 10-cm culture dishes. Stable CHO cell clones with or without AQP4 were then established for reference, see the previous paper [21]. Mixtures of stable CHO cells expressing either AQP4-IRES-EGFP or IRES-EGFP were cultured in a dish at a density of 1×10^5^ cells/dish at a ratio of 1∶0, 3∶1, 1∶1, 1∶3 or 0∶1 for a few days. CHO cells were seeded onto 60-mm dishes at a density of 1×10^5^ cells/dish and were transfected with ET_A_R-IRES-EGFP gene using Lipofectamine and plus reagents (Life Technologies), according to the manufacturer's instructions.

Primary cultured astrocytes derived form either WT- or AQP4-null mice (Acc. no. CDB0758K: http://www.cdb.riken.jp/arg/mutant%20mice%20list.html) were prepared as described previously [22]. This study was carried out in strict accordance with the recommendations in the guide for the care and use of laboratory animals of the MEXT of Japan. The protcol was approved by the Animal Care Committee of Keio University School of Medicine (Permit Number: 080007). All surgery were made under the protcol, it was performed to minimize suffering. The 5′- and 3′-homology arms were obtained from the BAC clone RP23-189N2 (BACPAC Resources).

Transgenic mouse embryonic stem (ES) cell lines with Tet-off system were cultured on the feeder dishes with conventional medium for undifferentiated-cell state, supplemented with 15% FCS, recombinant Leukemia inhibitory factor (LIF), 2-mercaptoethanol and so on as describe elsewhere. Doxycycline (Dox) and LIF (1 µg/ml) were added into the culture medium for the suppression of transgene and maintenance of undifferentiated cell state. For differentiation and transgene induction, cells were cultured in α-MEM medium without Dox and LIF. These differentiated cells were collected post 3day. Spontaneously differentiated ES cell were also harvested as a typical differentiated ES cells [18], [19].

### Plasmid construction

XhoI and SacII sites were added to rat endothelin receptor A (ET_A_R) cDNA [23] using PCR with the primers 5′-CTCGAGAAGATGGGTGTCCTTTGCTTTCTG-3′ and 5′-CCGCGGTTAGTTCATGCTGTCCTTGTGGC-3′. cDNA encoding the mouse AQP4 M1 isoform was connected in frame with the initiation codon of an internal ribosomal entry site (IRES-AQP4), and a NotI site was added at the 3′ end using 2-step PCR with the following primer sets: 5′-ACCGGACTCAGATCTCGAGCTCAAGCTTCG-3′ and 5′-CTCGCTGCAGCTCCGTCACTCATGGCCATATTATCATCGTG-3′; and 5′-CACGATGATAATATGGCCATGAGTGACGGAGCTGCAGCGAG-3′ and 5′-GCGGCCGCCTATACGGAAGACAATACCTC-3′. The PCR products were inserted into pGEM-T vector (Promega) to confirm their sequences. Then, the IRES-AQP4 cDNA was excised with HindIII and NotI and was inserted between the HindIII and NotI sites of a pIRES2-EGFP vector (Clonetech) to generate the pIRES-AQP4 vector. The pIRES-AQP4 vector was digested with XhoI and SacII, and the ET_A_R cDNA was inserted to produce the pET_A_R-IRES-AQP4 vector.

### Cryopreservation

Cultured cells were trypsinized, collected, and suspended in culture medium containing 10% FBS and cryoprotectant agent at a density of 1×10^6^ cells/mL for cryopreservation. In the cryopreservation, cryoprotectant agent, dimethylsurfoxide (DMSO) was used for final 10% concentration except for experiments regarding to a dose dependent effects of DMSO. The Bicell freezer box (Nihon Freezer) was provided at cooling rate of −1°C/min in a −80°C freezer. At cooling rates of −10, −30 and −50°C/min in mechanical freezers (Planer) were verified the monitoring of vapor phase and sample on the each cooling conditions. At the cooling rate of −120°C/min in a liquid nitrogen tank, frozen samples were directly immersing to liquid nitrogen. This condition was verified the monitoring of the temperature in the freeze preservation medium. All samples were frozen at each cooling rates of starting at room temperature, and finally stored in vapor phase nitrogen for a minimum 1 hour. Each experiment was repeated more than 3 times.

#### Viability assessment by Trypan blue staining

A trypan blue-exclusion assay was performed to determine cell viability at 0 h after thawing for each freezing condition. Samples stained with trypan blue were counted manually via light microscopy, and the post-thaw survival rate was calculated.

#### Viability assessment by colony-forming assay

The clonal growth ability of cells for each freezing rate was determined using a colony-forming efficiency (CFE) assay. Freezing/thawing cells (1×10^4^) were plated on 4-well chamber culture dishes and were cultured for three days (n>3). The colonies were fixed with ethanol, and the nuclei were stained using 4,6-diamidino-2-phenylindole (DAPI). The cell number was counted with DAPI in the visual filed of microscopy combination using MATLAB software (MathWorks Inc.).

#### Viability assessment by Flow-cytometery

Cell damage was examined using the PE Annexin V Apoptosis Detection Kit I (BD Biosciences), according to the manufacturer's instructions. Cell damage was measured using PE-Annexin V and 7-amino-actinomycin D staining. Live cells were both Annexin V and 7-AAD negative, cytoplasmic membrane-damaged cells were Annexin V positive and 7-AAD negative, and severe membrane damaged and dead cells were both Annexin V and 7-AAD positive. Cells were analyzed using a BD FACS Calibur™ flow cytometer. The rate of each condition of cells per 1×10^4^ cells was counted for a single panel.

#### Viability assessment by Scanning electron microscopy (SEM)

The morphologies of CHO cells before and after ultra-quick freezing were visualized using scanning electron microscope (SEM). Briefly, the cell suspension was first fixed in a fixative containing 2.5% glutaraldehyde/0.1 M PBS (Wako) at room temperature for 30 min. After washing twice with 0.1 M PBS, the cells were post-fixed with 1% osmium tetroxide (Wako) at room temperature for 30 min. The cells were then washed twice with PBS, dehydrated through serial gradients of ethanol (10 min for each gradient), and finally dried using a critical point dryer. The cells were placed on carbon to obtain a thin layer and then coated with osmium using plasma CVD equipment. Each sample was observed under a SEM (JCM- 5700; JEOL).

### Flow-cytometry

The numbers of AQP4-expressing cells before and after freezing were analyzed using a FACScalibur flow cytometer (Becton Dickinson) equipped with an argon laser emission of 488 nm, described in Miyazaki *et al.* [21]. AQP4-expressing cells were detected by staining with a monoclonal antibody against the extracellular domains of mouse AQP4 (1∶5) followed by the anti-mouse IgG conjugated with phycoerythrin (PE)(1∶100). A primary gate based on forward and side light scatters (FSC and SSC, respectively) was set to exclude dead cells or debris. The background level was estimated by omitting the primary antibody.

### Reverse transcriptase-polymerase chain reaction (RT-PCR) analysis

RT-PCR was performed to identify transcripts encoding mammalian AQPs in undifferentiated and differentiated mouse ES cells mouse. PCR-amplification was performed using specific primers for mouse AQP4: 5′-CTGGAGCCAGCATGAATCCAG -3′ and 5′-TTCTTCTCTTCTCCACGGTCA -3′ [24]. The experiments were carried out with a number of cycles that precedes saturation. PCR products (10 µl) were separated by electrophoresis on a 2.0% agarose gel and visualized after ethidium bromide staining under UV radiation. The expected PCR product size was 310 base pairs.

### Calcium imaging

Transfected pET_A_R-IRES-AQP4 cells with or without 3 cycles of freezing were cultured in 96-well thin-glass bottom dishes (1×10^4^ cells/well). The cells were loaded with 10 µM of the Ca^2+^ sensitive fluorescence indicator OregonGreen 488 BAPTA-1/AM (Life Technologies) at 37°C for 15 min in DMEM/F12 (1∶1) buffer (Life Technologies). Bolus injections of Oregon Green 488/AM were performed as described elsewhere [25]. The imaging of Oregon Green 488 fluorescence in cells was performed using a FV1000 confocal microscope equipped with a 60×/1.2 NA water-immersion objective (Olympus). Fluorescence data were analyzed using custom Fluoview software (Olympus). Ca^2+^ imaging was mediated using 10 µM of ET1 (Peptide Inc.) added 60 seconds later.

### Statistical analysis

Statistical analysis was performed using Microsoft Excel with the Statcel2 add-on (OMS). Data are presented as mean±S.D. The Student t-test was used for paired data of each of the groups compared (Statistical significance was defined as *P<0.05, **P<0.01, ***P<0.001).

## Results

### Freezing tolerance of mammalian cells expressing AQP

In this study, we evaluated possible roles of AQP in cryopreservation using mammalian cells, and its application in selecting a specific type of cells. Table 1 shows cell survival rates after ultra-quick freezing. Both, Chinese Hamster Ovary (CHO) and Madin-Darby canine kidney (MDCK) cells were affected by low survival rates of only 2.4±1.4% and 0.7±0.8%, respectively under cooling rate of −120°C/min (ultra-quick freezing) in 10% dimethyl sulfoxide (DMSO) solution. Interestingly, survival rates dramatically increased for cells stably expressing AQP4: 60.5±16.7% for AQP4-CHO cells and 37.2%±8.7% for AQP4-MDCK cells (Table 1). Cryoprotective effect was not limited to AQP4 since CHO cells expressing AQP1 also revealed high survival rate after ultra-quick freezing (Figure S1A). Surprisingly, survival rate of AQP4-CHO cells was still high even the concentration of DMSO is decreased from 10% to 1%. It should be noted that decreased concentrations of DMSO from 10% to 1% are associated with the decrease of osmolality from 1,600 mOsm to 432 mOsm (Figure S1B), which leads to decrease in osmotic stress and cellular toxicity [26], [27]. We further evaluated whether endogenously expressed AQP4 is sufficient to exert a cryoprotective effect, using primary cultured astrocytes derived from either wild-type or AQP4-null mice. Astrocytes from AQP4-null mice exhibited limited cell survival after ultra-quick freezing (2.5±2.3%), while the survival of wild-type astrocytes that express AQP4 endogenously, was significantly higher (54.1±6.4%) (Table 1).

**Table 1:** Cells expressing AQP either exogenously or endogenously are resistant to ultra-quick freezing/thawing.

Cell lines	Expression of AQP4	Cell viability ± S.D. (%)
CHO	(-)	2.4±1.4
	AQP4	60.5±16.7
MDCK	(-)	0.7±0.8
	AQP4	37.2±8.7
Astrocytes	AQP4 KO	2.5±2.3
	WT	54.1±6.4

Cell viability after ultra-quick freezing/thawing: comparison between CHO, MDCK cells and stably expressing AQP4 cells (AQP4-CHO, AQP4-MDCK), and also primary culture astrocytes from WT-mice (AQP4+) or AQP4-null mice (n ≧ 3, ***P<0.001).

### The effects of AQP on cell survival rate at different cooling rate

**Figure 1:**
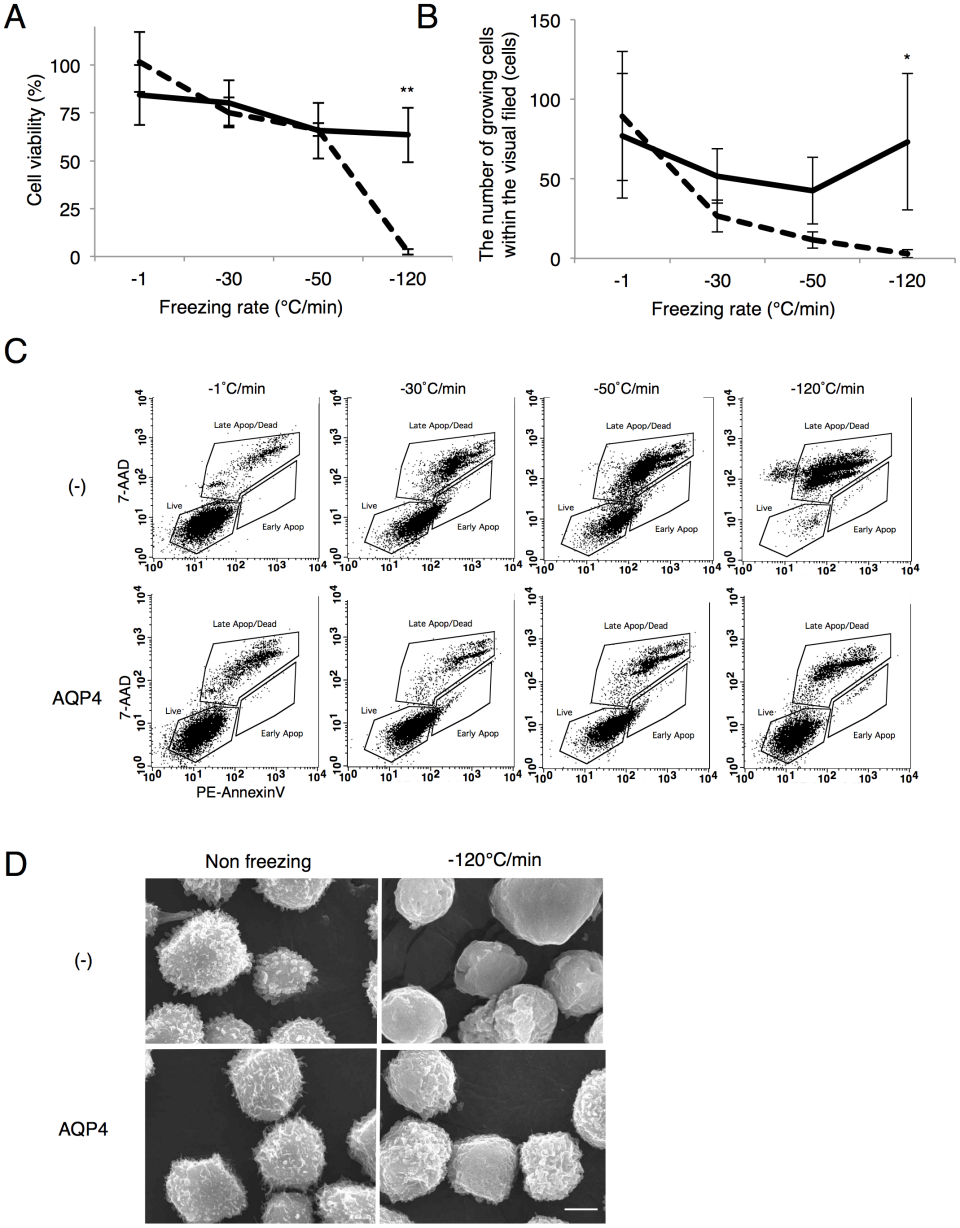
Freezing tolerance of cells expressing AQP after ultra-quick freezing. (A) Cell viability assessed using trypan blue-exclusion immediately at cooling rates of −1, −30, −50, and −120°C/min. The cell viability of control CHO cells is indicated by the dotted line, while that of AQP4-CHO cells is indicated by the solid line. Data are shown as the mean ± standard deviation (n ≧ 3, **P<0.01). (B) Colony-formation as assessed by counting the number of growing cells within the visual filed with DAPI staining at 3 days after freezing and thawing at cooling rates of −1, −30, −50, and −120°C/min. The cell viability of control CHO cells is indicated by the dotted line, while that of AQP4-CHO cells is indicated by the solid line. Data are shown as the mean ± standard deviation (n ≧ 3, *P<0.05). (C) Flow cytometry analyses of membrane damage in CHO cells (upper panels) and AQP4-CHO cells (lower panel) at the following freezing rates: −1, −30, −50, and −120°C/min. Cells were stained with phycoerythrin (PE)-conjugated Annexin V and 7-amino-actinomycin D (7-AAD) and were analyzed using flow-cytometry. Living cells were identified as cells with negative PE-Annexin V and 7-AAD staining (lower left region). Cytoplasmic membrane-damaged cells were Annexin V-positive (lower right region), whereas membrane damaged and dead cells were both Annexin V and 7-AAD-positive (upper right region). 1×10^4^ cells were analyzed by flow cytometry. (D) Scanning electron microscopy (SEM) study showing the surface characteristics of the membranes. Non-freezing (negative control, left panels) and ultra-quick freezing (−120°C/min, right panels) images show the morphological characteristics of CHO cells (upper panels) and AQP4-CHO cells (lower panels). Scale bar: 5 µm.

In order to understand the mechanisms behind the effects of AQP on cell survival after ultra-quick freezing, we next examined cell survival rate at different cooling rate. No significant difference in the cell viability was seen at freezing rates of −1, −30, or −50°C/min between the control CHO cells (dotted line) and those expressing AQP4 (AQP4-CHO cells) (solid line). However, at −120°C/min, a significant difference in survival rate was observed: 2.4±1.4% for CHO cells, and 60.5±16.7% for AQP4-CHO cells, indicating that the expression of AQP resulted in freezing tolerance at high cooling rate (Figure 1A).

A colony forming assay revealed an even clearer difference: significantly better survival rates were observed for AQP4-CHO cells at freezing rates of −30, −50, and −120°C/min. However, survival of CHO cells without AQP4 was not deteriorated by increasing freezing rates (Figure 1B).

To evaluate the cell membrane damages after ultra-quick freezing (−120°C/min), the cells were stained with phycoerythrin (PE)-conjugated Annexin V and 7-amino-actinomycin D (7-AAD) and then analyzed using flow-cytometry. The control CHO cells shifted to late apoptosis or cell death (both Annexin V and 7-AAD-positive) as freezing rate was increased, whereas AQP4-CHO cells remained viable for all tested freezing rates (Figure 1C). These results strongly suggest that the expression of AQP reduces membrane damage caused by freezing/thawing, thereby inhibiting cell death. Scanning electron microscope (SEM) imaging further confirmed the rescue of the cells from membrane damage after ultra-quick freezing by expression of AQP4. No difference in morphological characteristics (smooth and spherical cells with a slight bubbling effect) was observed between CHO cells and AQP4-CHO cells before freezing (Figure 1D, left panels). After ultra-quick freezing, CHO cells appeared to be burst, with flattering and invaginations caused by cell membrane damage, whereas AQP4-CHO cells exhibited spherically smooth surfaces, similar to cells before freezing (Figure 1D, right panels).

### Selection of cells expressing AQP by ultra-quick freezing/thawing

**Figure 2:**
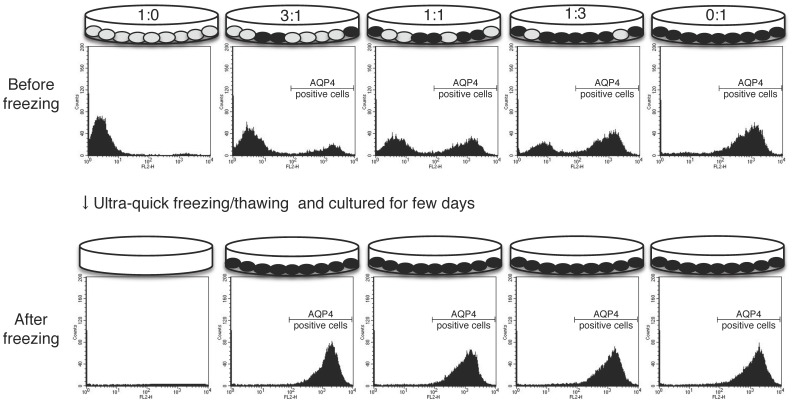
Selection of cells expressing AQP by ultra-quick freezing/thawing. CHO cells and AQP4-CHO cells were mixed in a ratio of 1∶0, 3∶1, 1∶1, 1∶3, or 0∶1, then cultured for a few days in the same culture dish. The schematic shows the mixed cell conditions for the CHO cells (empty circle) and the AQP4-CHO cells (filled circle). A flow-cytometry analysis shows the population of cells per 1×10^4^ cells from each culture dish before (upper panels) or after (under panels) ultra-quick freezing.

Having identified that AQP expression can acquire cryo-resistant feature of the cells, we hypothesize that cells expressing AQP can be selected by ultra-quick freezing. CHO cells (open circle) and AQP4-CHO cells (filled circle) were mixed in ratio of 1∶0, 3∶1, 1∶1, 1∶3, or 0∶1 and then cultured. The population of mixed-cell cultures before and after ultra-quick freezing was analyzed using flow-cytometry by staining with anti-AQP4 antibody followed by a secondary antibody conjugated with phycoerythrin (PE) (Figure. 2, upper panels). A clear shift in the PE intensity towards higher values occurred, regardless of the ratios of the mixture, indicating that only the AQP4-CHO cells were retained after ultra-quick freezing/thawing (Figure 2, lower panels). These data indicate that outstanding selection of cells can be achieved by ultra-quick freezing based on the AQP expression profile.

### Cell viability of undifferentiated and differentiated ES cells after ultra-quick freezing/thawing

**Figure 3:**
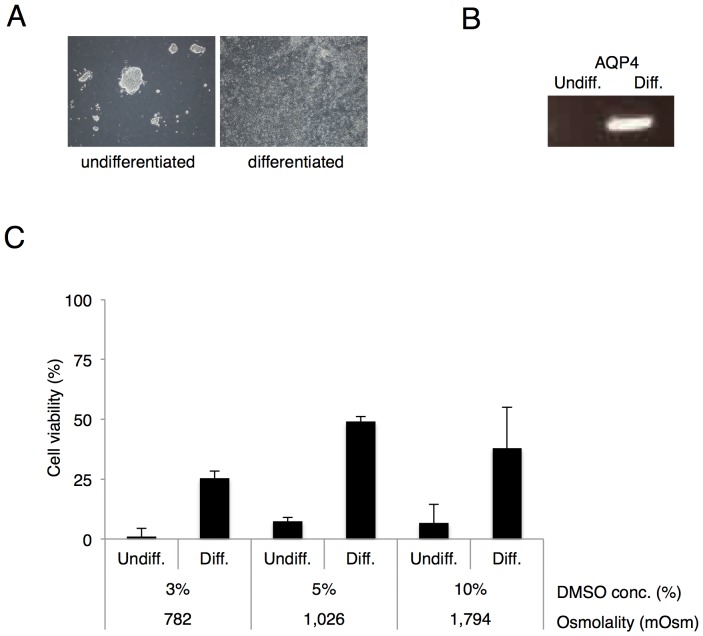
Cell viability of undifferentiated and differentiated ES cells after ultra-quick freezing/thawing. (A) Morphological assessment of the undifferentiated or the differentiated Ascl1 inducible ES cells (mouse ES cells under Tet-off system). (B) AQP4 mRNA expression in undifferentiated (Undiff.) or differentiated (Diff.) ES cells, assessed by RT-PCR. (C) Cell viability of the undifferentiated or the differentiated ES cells at different concentration of DMSO (3, 5 and 10%) after ultra-quick freezing. Data are shown as the mean ± standard deviation (n = 2–3, *P<0.05).

Bioinformatics approach revealed that AQP4 expression is gradually increased during ES cell differentiation into neural ectoderm lineage triggered with *Achaete-scute homolog1* (*Ascl1*) induction in Tet-off system and differentiated cells were confirmed by morphological changes of the cells (Figure 3A). To evaluate freeze tolerance along with AQP4 expression, we examined the viability of *Ascl1* induced cells after ultra-quick freezing. The expression of AQP4 mRNA was detected in the differentiated but not in the undifferentiated ES cells (Figure 3B). Then we examined cell viability of both undifferentiated and differentiated ES cells after ultra-quick freezing at different concentration of DMSO. Cell viability was 1.0±1.4% or 6.8±2.1% for the undifferentiated cells and 25.5±2.1% or 38.0±12.6% for the differentiated cells in media containing 3% or 10% DMSO medium, respectively (Figure 3C). These results strongly suggest that differentiated ES cells, if they express AQP4, can be highly selected since undifferentiated ES cells are more efficiently excluded by ultra-quick freezing with medium containing 3% DMSO comparing to 10% DMSO.

### Selection and concentration of CHO cells transiently transfected AQP4 by multiple cycles of ultra-quick freezing/thawing

We next examined if this method of selection can be applied to a transient expression system [28]. CHO cells were transiently transfected with a plasmid containing AQP4 and EGFP cDNAs connecting with internal ribosome entry site (IRES) (Figure S3A). EGFP was used to monitor the transfection efficiency. The transfection efficiency was significantly increased by ultra-quick freezing (61.3±7.3%), compared to cells that were not frozen (13.7±10.3%), 2 days after transfection (Figure S3B). This effect was transient, since the number of GFP positive cells gradually decreased and disappeared after 5 days post thawing (Figure S3C, blue and red line). However, we found that the population of GFP positive cells remained high even 5 days post thawing when we performed more than three cycles of freezing/thawing, suggesting that stably transfected cell line can be mimicked by this strategy (Figure S3C, green and purple line). We noticed that the order of insertion of cDNA in the IRES construct is also important [29] since flow-cytometry analyses revealed different transfection efficiency assessed with first gene (AQP4) or second gene (EGFP), 73.0±4.7% and 52.8±5.0%, respectively after 3^rd^ ultra-quick freezing/thawing (Figure S3D). IRES has been widely used to translate multiple gene products from a transcript and little known the efficiency of IRES-dependent second gene expression relative to first gene expression. In addition, the latter part is sometimes truncated when the construct is integrated into the genomic DNA of host cells. Our results showed the reduced expression of the second gene in pAQP4-IRES-EGFP (Figure S3D). It is, therefore, important to insert the gene of interest as the first gene and AQP as the second gene in the IRES construct for this purpose.

### Functional assay of the target gene transiently co-transfected with AQP4

**Figure 4:**
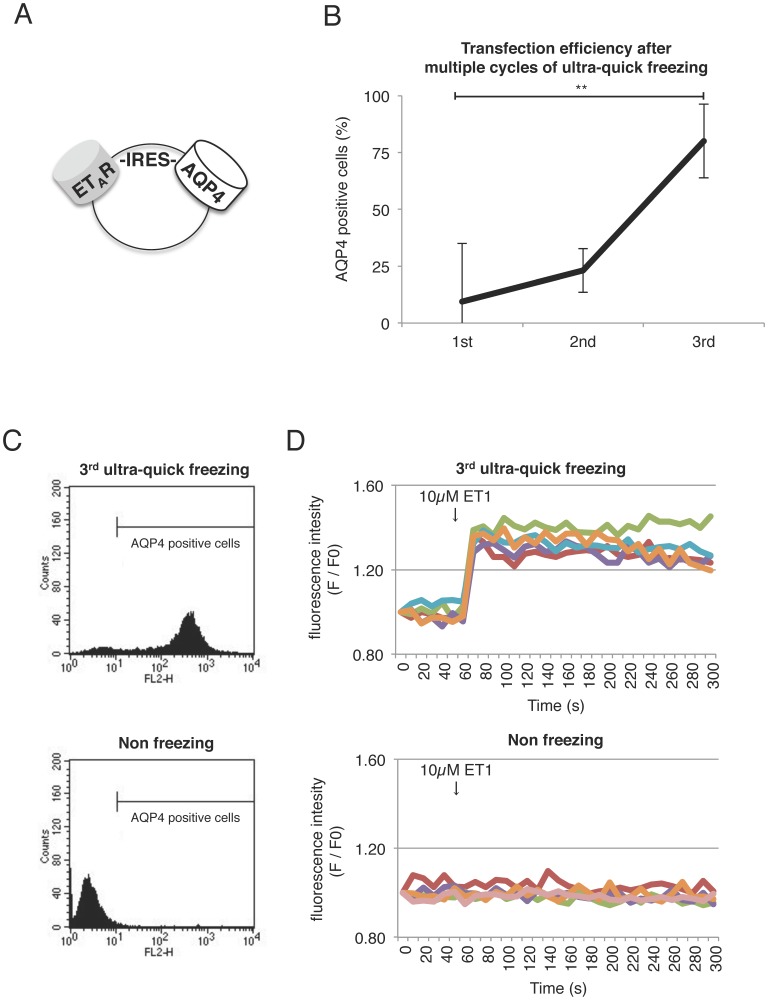
Functional assay of the target gene transiently co-transfected with AQP4 after multiple-cycles of ultra-quick freezing/thawing. (A) Plasmid containing ET_A_R-IRES-AQP4 cDNA. (B) The increased transfection efficiency in CHO cells transiently transfected with ET_A_R-IRES-AQP4 by multiple cycles of ultra-quick freezing/thawing. Data are shown as the mean ± standard deviation (n ≧ 3, **P<0.01). (C) Flow-cytometry analyses quantitatively show the population of AQP4 positive cells after 3^rd^ ultra-quick freezing (top) or without any freezing (bottom). (D) Intracellular calcium imaging in response to endothelin-1 (ET_1_). Cells were pre-loaded with the calcium indicator Oregon Green/AM. Intensity versus time traces of the cells is shown after 3^rd^ ultra-quick freezing (top) or without freezing (bottom). Five representative traces out of 20 measurements are shown for each group. See also Video S1 and S2.

To examine if this method can be applied for a functional assay of the gene of interest, a plasmid containing ET_A_R (Endothelin Receptor A)-IRES-AQP4 construct was transiently transfected into CHO cells (Figure 4A). We performed functional assay for ET_A_R in the AQP4-positive cells concentrated after multiple ultra-quick freezing/thawing or cultured post transfection without freezing. We confirmed that the cell survival rate significantly increased by transfecting the ET_A_R-IRES-AQP4 construct and stably retained high values after the third freezing/thawing and during three weeks after transfection (Figure 4B). A flow-cytometry analysis further confirmed that most of the cells were AQP4-positive (Figure 4C). We next performed Ca^2+^ imaging to assess the function of ET_A_R. The [Ca^2+^]i increases, evoked by stimulation with endothelin-1 (ET-1), were almost negligible in the non-freezing cells. In contrast, the [Ca^2+^]i increases were clearly observed in treated cells (91%) even three weeks later (Figure 4D and Video S1). These results strongly suggest that multiple cycles of ultra-quick freezing/thawing episodes not only increase the transfection efficiency of the genes co-transfected with AQP, but also allow the cells to stably express them.

## Discussion and Conclusions

Here we present a novel and efficient method for selecting or concentrating mammalian cells based on our findings that cells expressing AQPs acquired tolerance to ultra-quick freezing by evading cell membrane damages. During freezing/thawing, cells are exposed to a variety of stresses, such as changes in temperature, changes in water content, ice crystal formation, and changes in solute concentration [1]–[5]. At low cooling rates, ice crystal formation remains extracellular whereas, at high cooling rates, extensive intracellular super-cooling and the formation of intracellular ice crystals occur, causing cellular injury to the plasma membrane [4], [5]. Consistently, CHO cells survive after freezing at low cooling rates but died at high cooling rates or ultra-quick freezing. Thus, the finding that the expression of AQP rescues cells from membrane damage and significantly improves cell survival rate after ultra-quick freezing is remarkable. The freezing tolerance of cells depends on membrane water permeability and the dynamics of water molecules inside and outside of cells. AQP-mediated facilitated diffusion of water molecules is temperature independent [30], whereas the simple diffusion of water though a lipid bilayer depends on temperature, implying that the difference in water permeability becomes more obvious at lower temperatures; the water permeability of a cell membrane without AQP quickly becomes limited, whereas that of a membrane containing AQP remains relatively high. We therefore suspect that AQP can contribute to the improvement in cryopreservation by increasing membrane water permeability even under low temperatures [5], [30]. Since this unique feature of AQP can be seen not only for exogenous AQP expression but also for endogenous AQP expression, diverse applications of this selection system, from basic science to clinical medicine including regenerative and reproductive medicine, are feasible [5], [27].

The clinical application of iPS cells or ES cells is currently one of the most urgent issues in biology and medicine [31]. Once iPS cells or ES cells are differentiated into tissues with proper functions, undifferentiated cells must be removed to avoid a potential risk of cancer [32], [33]. Since cryopreservation is an important and necessary step proceeding the administration of cells and tissues to patients, our selection system based on ultra-quick freezing could be easily and widely used without requiring any additional procedures [9], [10]. Bioinformatics is a powerful method of identifying the gene expression profile at each stage of differentiation in ES or iPS cells [18], [19]. Once the expression of AQPs in cell lineages or cell types of interest have been identified, these cells could be isolated using ultra-quick freezing. For example, since AQP4 is exclusively expressed into neuro-ectoderm lineage and neural stem cells [18]–[20], it may be possible to isolate neural stem cells using this method (Figure S2A and B).

Interestingly, multiple freezing/thawing steps can further concentrate the transfected cells continuously, thereby mimicking stable cell lines. All cells transfected with an expression construct containing AQP4 are initially resistant to ultra-quick freezing and can survive after the first thawing. However, most of the plasmids incorporated into the cells independently localize in the nucleus, and will not be amplified because in most cases, a plasmid has no origin for replication in the mammalian cells, thereby the cells are gradually lost the expression of the plasmids as the cells proliferate. Loss of the plasmids makes cells negative for a gene of interest as well as AQP and sensitive to ultra-quick freezing/thawing. Thus, cells lacking the plasmid will be removed in the process of multiple freezing/thawing. On the other hand, once the construct is integrated into the genomic DNA of host cells, it can be replicate in synchronization with replication of the genomic DNA, resulting in accumulation of cell expressing a gene of interest. Our protocol can achieve concentration of cells with relatively higher expression level of a gene of interest in a shorter period than conventional methods probably because a threshold of expression level of AQP4 required for cell survival is high as compared with that of drug-resistant genes such as the neomycin-resistant gene. There is some concern that exogenous expression of AQP might affect the functions of genes that are co-transfected as targets of investigation, but significant effects are unlikely, based on the integrity of ET_A_R function observed in the present study, although the possibility of interference should always be carefully evaluated.

Finally, We would like to point out that this method works even under conditions that reduce the concentration of cryoprotectants and the osmotic stress. This feature has important advantages for clinical application since a high concentration of DMSO can damage cells, and since complicated thawing procedures required for cryopreservation can be avoided [10], [26], [27].

Taken together, the above findings suggest that an efficient and safe cell-selection system combining AQP expression and ultra-quick freezing could be used as a novel method for selecting or concentrating cells for diverse purposes from basic to clinical applications.

## Supporting Information

Figure S1 **Exogenously cells expressing AQP are resistant to ultra-quick freezing/thawing.** Cell viability after ultra-quick freezing/thawing, comparing between (A) CHO cells and CHO cells stably expressing AQP1 (AQP1-CHO cells). Data are shown as the mean ± standard deviation (n ≧ 3, ***P<0.001) (B) Cell viability of CHO cells or AQP4-CHO cells under different concentrations of DMSO (1% to 10%). Data are shown as the mean ± standard deviation (n ≧ 3, ***P<0.001).
(TIFF)

Figure S2 **Bioinformatics indicating the AQP4 expression during the differentiation of ES cells into different cell lineages.** (A) Global gene expression profiles of 27 different cell types. Principal component analysis (PCA) shows that individual cell types are mapped in the 3D space according to the first three principal components (PC1, PC2 and PC3). Cell lineages with post-differentiated days are indicated as: light green and green for endoderm, marine blue for trophectoderm, orange for neuro-ectoderm and red for neural stem and progenitor cells. This supplement figure S2A was modified from a reference paper [19]. (B) Microarray data indicating the expression patterns during differentiation of ES cells into different cell lineages. The Y-axis indicates AQP4 gene expression (log intensity), and the X-axis indicates the differentiation of ES cells into multiple cell lineages (lineage and post-differentiated days). Cell lineages are indicated with light green and green for endoderm, marine blue for trophectoderm, orange for neuro-ectoderm and red for neural stem and progenitor cells. This indicates the increased expression of AQP4 during the differentiation of ES cells into neuro-ectoderm as well as neural stem and progenitor cells (the NIA Array Analysis software) [19].
(TIFF)

Figure S3 **Increased transfection efficiency and stability of CHO cells transfected with AQP4-IRES-EGFP after multiple cycles of ultra-quick freezing/thawing.** (A) Schematic drawing of a plasmid containing AQP4-IRES-EGFP gene. (B) Impact of ultra-quick freezing on transfection efficiency that was assessed with GFP positive cells. Data are shown as the mean ± standard deviation (n ≧ 3, **P<0.01) (C) The number of GFP positive cells after multiple cycles of freezing/thawing (Blue line: 1^st^, red line: 2^nd^, green line: 3^rd^ and purple line: 4^th^ freezing/thawing) as indicated at 1, 3 and 5 post-thawing days. (D) The order of insertion of cDNA in the IRES construct affected transfection efficiency, which was assessed with first gene (AQP4) or second gene (EGFP), 73.0±4.7% and 52.9±5.0%, respectively after 3^rd^ ultra-quick freezing/thawing. Data are shown as the mean ± standard deviation (n ≧ 3, **P<0.01).
(TIFF)

Video S1 **Time-lapse imaging of ET_1_-induced intracellular calcium with 3 cycles of ultra-quick freezing/thawing.** CHO cells were transiently transfected with ET_A_R-IRES-EGFP. It was assessed after 3 cycles of ultra-quick freezing/thawing (see Figure 4D, top). Intracellular calcium imaging in response to endothelin-1 (ET_1_). The cells were pre-loaded with the calcium indicator Oregon Green/AM (see Materials and Methods). Calcium imaging is shown using a video obtained after 3^rd^ ultra-quick freezing (Video S1). Bar, 20 µm. Frame size is 320×320 µm.
(AVI)

Video S2 **Time-lapse imaging of ET_1_-induced intracellular calcium without any freezing.** CHO cells were transiently transfected with ET_A_R-IRES-EGFP. It was assessed without any freezing (see Figure 4D, bottom). Calcium imaging is shown using a video obtained the non freezing (Video S2). Bar, 20 µm. Frame size is 320×320 µm.
(AVI)
