# Supplementary figures and images for: Unprecedented Cell-Selection Using Ultra-Quick Freezing Combined with Aquaporin Expression
Source: PLoS One. 2014 Feb 18;9(2):e87644. doi: 10.1371/journal.pone.0087644 (PMC3928110; doi:10.1371/journal.pone.0087644)

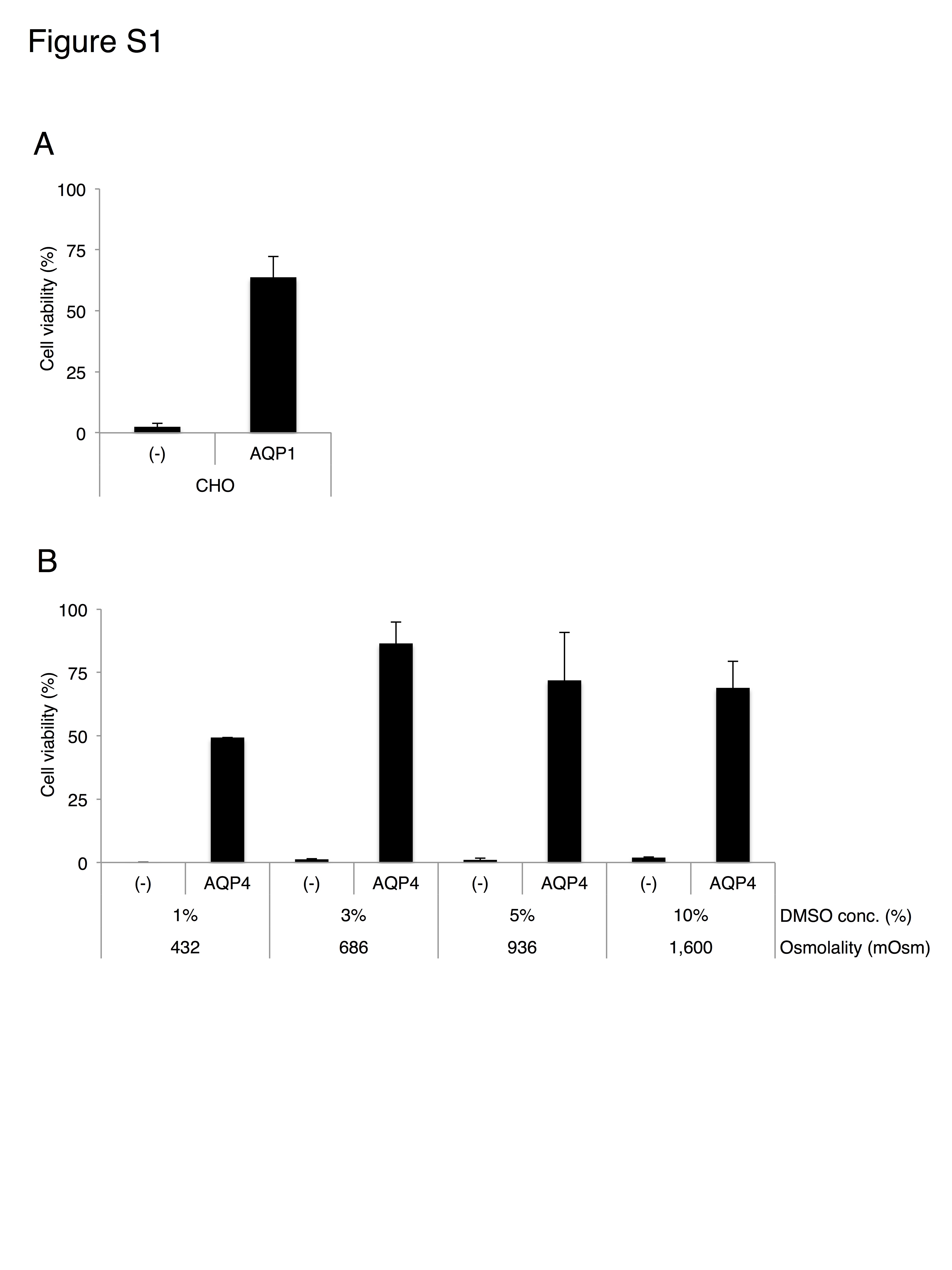

Supplement: Figure S1 — Exogenously cells expressing AQP are resistant to ultra-quick freezing/thawing. Cell viability after ultra-quick freezing/thawing, comparing between (A) CHO cells and CHO cells stably expressing AQP1 (AQP1-CHO cells). Data are shown as the mean ± standard deviation (n ≧ 3, ***P<0.001) (B) Cell viability of CHO cells or AQP4-CHO cells under different concentrations of DMSO (1% to 10%). Data are shown as the mean ± standard deviation (n ≧ 3, ***P<0.001). (TIFF) [file pone.0087644.s001.tiff]

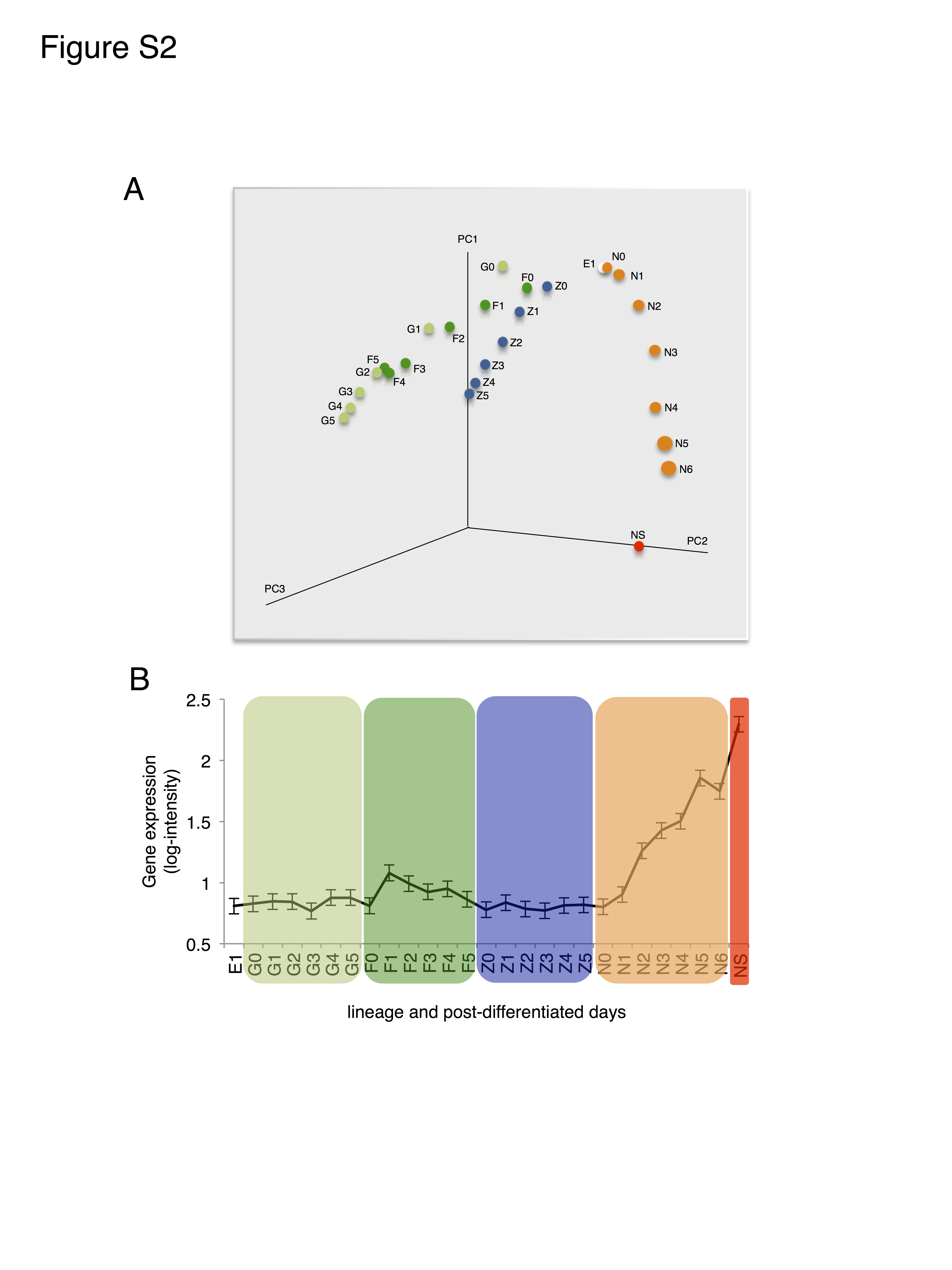

Supplement: Figure S2 — Bioinformatics indicating the AQP4 expression during the differentiation of ES cells into different cell lineages. (A) Global gene expression profiles of 27 different cell types. Principal component analysis (PCA) shows that individual cell types are mapped in the 3D space according to the first three principal components (PC1, PC2 and PC3). Cell lineages with post-differentiated days are indicated as: light green and green for endoderm, marine blue for trophectoderm, orange for neuro-ectoderm and red for neural stem and progenitor cells. This supplement figure S2A was modified from a reference paper [19]. (B) Microarray data indicating the expression patterns during differentiation of ES cells into different cell lineages. The Y-axis indicates AQP4 gene expression (log intensity), and the X-axis indicates the differentiation of ES cells into multiple cell lineages (lineage and post-differentiated days). Cell lineages are indicated with light green and green for endoderm, marine blue for trophectoderm, orange for neuro-ectoderm and red for neural stem and progenitor cells. This indicates the increased expression of AQP4 during the differentiation of ES cells into neuro-ectoderm as well as neural stem and progenitor cells (the NIA Array Analysis software) [19]. (TIFF) [file pone.0087644.s002.tiff]

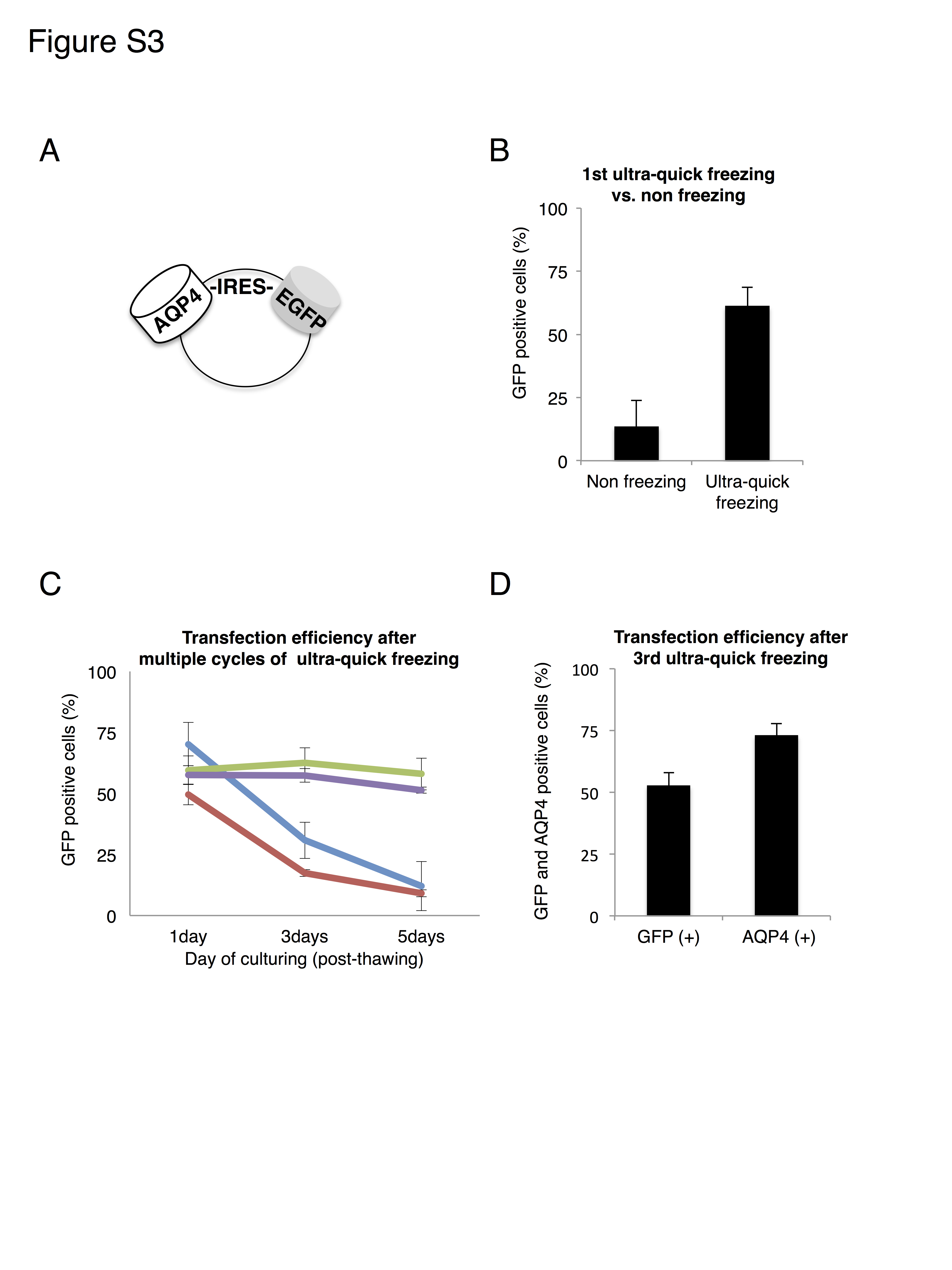

Supplement: Figure S3 — Increased transfection efficiency and stability of CHO cells transfected with AQP4-IRES-EGFP after multiple cycles of ultra-quick freezing/thawing. (A) Schematic drawing of a plasmid containing AQP4-IRES-EGFP gene. (B) Impact of ultra-quick freezing on transfection efficiency that was assessed with GFP positive cells. Data are shown as the mean ± standard deviation (n ≧ 3, **P<0.01) (C) The number of GFP positive cells after multiple cycles of freezing/thawing (Blue line: 1st, red line: 2nd, green line: 3rd and purple line: 4th freezing/thawing) as indicated at 1, 3 and 5 post-thawing days. (D) The order of insertion of cDNA in the IRES construct affected transfection efficiency, which was assessed with first gene (AQP4) or second gene (EGFP), 73.0±4.7% and 52.9±5.0%, respectively after 3rd ultra-quick freezing/thawing. Data are shown as the mean ± standard deviation (n ≧ 3, **P<0.01). (TIFF) [file pone.0087644.s003.tiff]
